# Characterization of the polysaccharide from *Bletilla striata* and its inhibitory effects on amylolytic enzymes and prebiotic activity

**DOI:** 10.3389/fnut.2025.1625260

**Published:** 2025-07-25

**Authors:** ChunYan Fu, Xuefeng Deng, Jun Gao

**Affiliations:** College of Sports Science, Harbin Normal University, Harbin, China

**Keywords:** *Bletilla striata* polysaccharide, characterization, hypoglycemic function, prebiotics, diabetes

## Abstract

In the current study, a new polysaccharide named Bletilla striata polysaccharide (BSP) with an average molecular weight (*M*_w_) of 85.4 kDa was obtained from *B. striata.* The inhibitory effects of BSP on α-amylase and α-glucosidase, as well as its prebiotic properties, were determined. The results showed that BSP was a neutral polysaccharide and is composed of mannose, glucose, and galactose with a relative molar ratio of 3.2:5.4:1.0. The main sugar residues in BSP were →4)-α-D-Glc*p*-(1→, →6)-β-D-Gal*p*-(1→, →4)-β-D-Man*p*-(1→, and β-D-Man*p*-(1→. BSP displayed strong inhibitory effects against α-amylase and α-glucosidase and exhibited competitive inhibitory kinetics. At 4.0 mg/mL, the inhibitory rates of 67.75 ± 0.45% on α-amylase and 48.24 ± 1.02% on α-glucosidase were obtained, respectively. Moreover, BSP can serve as a carbon source to facilitate the proliferation of probiotics. These findings support the potential application of BSP in diabetes management.

## 1 Introduction

The high incidence of diabetes has become a pressing problem to be solved. By 2024, the number of adult diabetic patients in China was approximately148 million (data from the International Diabetes Federation). This disease has seriously threatened the quality of life and safety of people (1). There may be many causes of diabetes; among them, the intake of starchy foods is a common factor for elevated blood sugar. It is well known that α-amylase and α-glucosidase are two key enzymes during the hydrolysis of dietary starch (2), inhibiting these two enzymes can reduce the starch breakdown and intestinal absorption, resulting in the decrease of postprandial hyperglycemia (3, 4). Therefore, the effective inhibitors of these two enzymes can be used as a treatment method for type 2 diabetes.

*Bletilla striata*, a species of the orchidaceae family, has been used as a traditional Chinese herbal medicine for many years (5). The rhizomes of *B. striata* can be used to treat hematemesis, hemoptysis, traumatic hemorrhage, and ulcerative carbuncles. Many compounds, such as various organic acids, polysaccharides, and dihydrophenanthrenes, have been isolated from *B. striata.* Among all the active compounds, the polysaccharides were the major active components responsible for their various biological effects (6, 7). For example, He et al. reported that the *B. striata* polysaccharides (BSPs) showed an inhibitory action against *Escherichia coli*, *Staphylococcus aureus*, *Bacillus subtilis*, and *Aspergillus niger* and presented a dose-dependent manner in the tested concentrations (8). Chen et al. found that BSPs could alleviate midgut epithelium damage by increasing glutathione-S-transferase and superoxide dismutase (SOD) activities in mercury-induced *Drosophila* (9). Xu et al. reported that BSPs could encourage wound healing through vascular regeneration and collagen deposition (10). Moreover, low molecular weight was a characteristic feature of BSPs. For instance, Chen et al. prepared a polysaccharide from *B. striata* with an average *M*_w_ of 9.1 × 10^4^ Da (11). Wang et al. extracted glucomannan (1.7 × 10^5^ Da) from *B. striata* using hot water at 80°C (12). Liu and Liu obtained an alcohol-soluble BSP with an average *M*_w_ of 2.29 × 10^4^ Da using cold water (13). The above studies demonstrated that BSPs with lower *M*_w_ exhibited superior biological activities and had potential applications as biological macromolecule materials in medicine, food, and cosmetics. However, reports on the hypoglycemic effects and the prebiotic activities of BSPs were scarce. Further research is needed to prove this potentiality.

The purpose of this paper is to conduct a preliminary study to extend the application of BSPs in the treatment of diabetes. First, a polysaccharide from BSP was obtained and characterized through ultraviolet spectroscopy (UV), Fourier transform infrared spectroscopy (FT-IR), nuclear magnetic resonance (NMR) spectroscopy, scanning electron microscopy (SEM), and the Congo red test. The inhibitory effects and mechanism of BSP against α-amylase and α-glucosidase were investigated; in addition, the prebiotic activities of BSP were also tested. The findings provide a scientific basis for expanding the research field and the functional utility of the polysaccharides from *B. striata*.

## 2 Materials and methods

### 2.1 Materials

The rhizomes of *B. striata* were purchased from Harbin Traditional Chinese Medicine Co., Ltd. (Heilongjiang, China). Diethylaminoethyl cellulose 52 (DADE-52) and Sephadex G-100 were purchased from Yuanye Biological Co. (Shanghai, China). Monosaccharide standards and dextran standards were purchased from Sangon Co. (Shanghai, China). Trifluoroacetic acid (TFA) was purchased from Sigma–Aldrich (Saint Louis, MO, United States). *p*-Nitrophenyl-β-D-glucopyranoside (pNPG), α-glucosidase (100 U/g), and α-amylase (3,700 U/g) were purchased from Beijing Solarbio Science & Technology Co., Ltd. (Beijing, China). *Lactobacillus plantarum* and *Lactobacillus acidophilus* were purchased from Beijing Kezhan Biotechnology Co., Ltd. (Beijing, China). All the reagents were of analytical grade.

### 2.2 Extraction of BSP

Hot water extraction was applied to prepare the *B. striata* polysaccharide, as described in the study by Chen et al. (11). Briefly, the dried *B. striata* powder (5 g) was dissolved in deionized water (250 mL) in a conical flask at 80°C and stirred for 120 min. Then, the extracts were centrifuged and deproteinized using Sevage reagent (chloroform: n-butanol = 4:1). Next, a precipitation procedure with 80% (v/v) ethanol was performed, and the crude polysaccharide was obtained after lyophilization.

### 2.3 Isolation and purification of the crude polysaccharides

The crude polysaccharide (10 mg/mL, 2 mL) was purified on a DADE-52 cellulose column (2.0 *×* 40 cm) and eluted with deionized water at a flow rate of 1.0 mL/min. The eluate was collected, and its polysaccharide content was detected by the phenol-sulfuric acid method (14). Subsequently, the sample was further separated on a Sephadex G-100 column (2.0 × 30 cm) using deionized water as eluant at a flow rate of 0.5 mL/min; the top peak of the elution fraction was collected, lyophilized, and labeled as BSP.

### 2.4 Characterization of BSP

#### 2.4.1 Chemical composition

The chemical components, including total sugar, uronic acid, and protein, were determined according to the published methods (14–16).

#### 2.4.2 *M*_w_

The *M*_w_ of BSP was measured by gel permeation chromatography (WATERS-2695-2414, Waters Corporation, Milford, MA, United States) equipped with a TSKgelGMPWXL column (7.8 × 300 mm) (TOSOH Biotechnology Co., Ltd. Shanghai, China) and an RID-20 refractive index detector (Shimadzu, Japan). A 10-μL BSP solution (2 mg/mL) filtered through a 0.45-μm membrane was loaded on the instrument with deionized water (0.1% NaNO_3_) as the eluent at 35°C. The standard curve was obtained based on T-series dextrans (T-5, T-10, T-40, T-70, and T-110) (lg*M*_w_ = −0.48 *t* + 8.91, *R*^2^ = 0.9958).

#### 2.4.3 Monosaccharide composition

Referencing the method in a prior study, the monosaccharide composition of BSP was analyzed (17). BSP was hydrolyzed with a 2 mol/L HCl solution and then derivatized using PMP before detection by high-performance liquid chromatography (WATERS-2695-2414, Waters Corporation, Massachusetts, United States). D-glucose, D-mannose, D-galactose, L-arabinose, D-xylose, and L-rhamnose were used as standards using the same assay method. The monosaccharide composition was determined by comparing retention time and peak area.

#### 2.4.4 Ultraviolet (UV) and FT-IR spectra

The UV spectrum of the BSP solution (1.0 mg/mL) was determined using a double-beam UV spectrophotometer (Helios Gamma, Thermo Corporation, Wales, England) in the range of 200–500 nm.

The dried BSP powder was mixed with KBr and ground into flakes. A Fourier transform infrared spectrometer (ALPHA-T, Bruker Co., DE, Germany) was used to record the spectra in the range of 400–4,000 cm^−1^ with a resolution of 4 cm^−1^ and an accumulation of 32 scans.

#### 2.4.5 Methylation analysis

Methylation of BSP was carried out according to the method of Ciucanu and Kerek (18). Then, the methylated polysaccharide was hydrolyzed with 2 mol/L TFA at 100°C for 6 h, and excess acid was removed by evaporation. The hydrolysates were reduced with NaBH_4_ for 24 h and acetylated with acetic anhydride-pyridine (1:1) at 100°C for 2 h. These acetates were analyzed with gas chromatography–mass spectrometry (GC–MS) (Varian-450-GC-320-MS, Agilent Co., Massachusetts, United States) using an HP-5 MS fused silica capillary column (30 m × 0.25 mm, 0.25 μm, Agilent, NY, United States). The parameters were set as follows: the injection temperature was 220°C, the initial temperature was 160°C for 2 min, followed by 5°C/min to 210°C for 1 min, and finally 10°C/min to 260°C for 10 min.

#### 2.4.6 NMR analysis

BSP was dissolved in D_2_O with a concentration of 20 mg/mL. Proton nuclear magnetic resonance (^1^H NMR) and carbon-13 nuclear magnetic resonance spectroscopy (^13^C NMR) spectra were determined using a Bruker DRX-600 NMR spectrometer (Bruker, Germany) at 25°C.

#### 2.4.7 Congo red test

First, the BSP solution (2.0 mg/mL, 2.0 mL) and the Congo red solution (80 μmol/L, 2.0 mL) were mixed, and the NaOH solution (1.0 mol/L, 0.0–4.0 mL) was separately added into the mixture. The λmax of the mixed solution was then tested by a UV spectrometer in the range of 400–700 nm.

#### 2.4.8 Iodine–potassium iodide (I_2_–KI) test

The BSP solution (1.0 mg/mL, 1.0 mL) was added to the iodine reagent (0.5 mL, containing 0.2% KI and 0.02% I_2_, w/v), and the mixture was stirred for 10 min. The absorbance, from 300 to 700 nm, was determined using an Agilent 8453 G1103A ultraviolet–visible (UV–Vis) spectrophotometer (Beijing Purkinje General Instrument Co., Beijing, China).

#### 2.4.9 SEM

Appropriate amounts of BSP powders were glued onto the sample stage and plated with a gold film using the sputtering method. The morphological features were observed using a scanning electron microscope (Thermo Fisher Scientific Inc., Waltham, MA, United States) at an accelerating voltage of 5.0 kV.

#### 2.4.10 Atomic force microscopy

The BSP solution (5.0 μg/mL, 10.0 μL) was deposited onto the mica slice and allowed to dry naturally. Then, the atomic force microscopy (AFM) image was obtained using a Dimension FastScan (Bruker, Germany) at a scanning range of 5 μm × 5 μm and a frequency of 1 Hz.

### 2.5 Measurement of inhibitory action of BSP on α-amylase and α-glucosidase

Based on the reported literature (19), the inhibitory effects of BSP at different concentrations (0.1–4.0 mg/mL) on the two enzymes were tested. The inhibitory rate was calculated as follows:


$$\mathrm{IR}=(1-\frac{A_{a}-A_{b}}{A_{c}-A_{d}})\times 100\%,$$

where *A*_a_ is the solution absorbance containing enzyme, substrate (starch or pNPG), and BSP; *A*_b_ is the solution absorbance containing substrate (starch or pNPG), and BSP; *A*_c_ is the solution absorbance without BSP, and *A*_d_ is the solution absorbance in the absence of the enzyme and BSP.

### 2.6 Inhibition kinetics determination of BSP on α-amylase and α-glucosidase

#### 2.6.1 α-amylase inhibition kinetics

First, each 1.0 mL BSP solution (0.0, 1.0, 2.0, and 4.0 mg/mL) was mixed with 1.0-mL α-amylase solution (0.0, 0.2, 0.4, 0.6, and 0.8 mg/mL) and stirred for 5 min at 37°C; then, the starch solution (1.0 mL, 2.0 mg/mL) was poured to the mixture. After a 30-min reaction, 2.5-mL of dinitrosalicylic acid was added to the mixture, and the reaction was terminated by incubation in boiling water for 5 min. During the reaction, the absorbance of the solution was continuously tested at 540 nm at 25°C. The reaction rate *v* (Δ*A*/min) is calculated according to the change value of the linear change of absorbance over time divided by time, with the enzyme concentration as the abscissa and the reaction rate as the ordinate to determine the inhibition type of polysaccharide on α-amylase activity.

The type of reversible inhibition of α-amylase activity was determined. The α-amylase solution (0.6 mg/mL) was mixed with a 1.0-mL BSP solution (0.0, 1.0, 2.0, and 4.0 mg/mL) and then incubated at 37°C for 5 min. Next, a 1.0-mL starch solution (0.5, 1.0, 1.5, 2.0, and 2.5 mg/mL) was added to the mixture. Using the method described above, the absorbance was tested at 540 nm every 2 min. The Lineweaver–Burk curve, using 1/[S] as the abscissa and 1/*v* as the ordinate, determines the type of reversible inhibition of α-amylase and calculates the enzyme inhibition constant *K*_i_.

#### 2.6.2 α-glucosidase inhibition kinetics

The BSP solution (40 μL; 0.0, 0.5, 1.0, and 2.0 mg/mL) and pNPG solution (20 μL, 2.0 mmol/L) were added sequentially to 96-well plates, shaken for 1 min, and incubated at 37°C for 5 min. Then, the α-glucosidase solution (40 μL, 0.0–0.16 U/mL) was added to the mixture. Next, the 96-well plate was put into a microplate reader (Spark 10 M, Tecan Group Ltd., Switzerland). The initial velocity (*v*) was determined by monitoring the absorbance at 405 nm every 1 min. The inhibition (reversible or irreversible type) was confirmed by plotting *v* against [E].

Reversible inhibition was further studied. The BSP solution (40 μL, 0.0–2.0 mg/mL) and the α-glucosidase solution (40 μL, 0.16 U/mL) were added to 96-well plates, shaken for 1 min, and incubated at 37°C for 5 min. The pNPG solution (20 μL, 0.25–4.0 mmol/L) was added to the mixture. Following the same operation as described above, the absorbance was recorded every 1 min, and the Lineweaver–Burk curves were obtained.

### 2.7 Prebiotic activities determination of BSP

#### 2.7.1 Preparation of bacterial strain

The *L. plantarum* and *L. acidophilus* strains were activated using modified MRS medium base according to the reported methods (20). Carbohydrate-free MRS broth supplemented with 0.1% (w/v) BSP was used as the basal medium to investigate the proliferation of bacterial strains. Glucose was used as the positive control. The active *L. plantarum* and *L. acidophilus* strains were separately transferred to the MRS broth medium at a concentration of 1 × 10^6^ CFU/mL and then incubated in an anaerobic chamber at 37°C for 48 h under anaerobic conditions (85% N_2_, 10% CO_2_, and 5% H_2_).

#### 2.7.2 Effects of BSP on the growth of probiotics

The proliferation of the two strains in the media was determined by measuring the optical density at 600 nm at intervals of 12, 18, 24, and 30 h during the incubation period. The pH of the medium was determined using a pH meter (Thermo Fisher Scientific (China) Co., Ltd, Beijing, China) at 6 h intervals.

#### 2.7.3 Analysis of surface morphology of the bacteria by SEM

After 30 h incubation in BSP medium, the probiotics were centrifuged, washed twice with 0.9% NaCl, and then fixed with 2% glutaraldehyde. The morphology of the probiotics was observed using SEM at a 20.0 kV acceleration voltage.

#### 2.7.4 Measurement of short-chain fatty acids

After 30 h incubation, the levels of short-chain fatty acids (SCFAs) in the medium were analyzed for acetic acid, propionic acid, and butyric acid using GC–MS (7890 GC system equipped with 7000C Triple Quad MSD, Agilent) equipped with an Agilent DB-FFAP column (30 m × 0.25 μm). Helium was used as the carrier gas. The inlet temperature was 230°C. The column temperature was improved from 90°C to 120°C at 8°C/min, then to 150°C at 5°C/min, and finally to 250°C at 25°C/min and held for 5 min.

### 2.8 Statistical analysis

All experiments were performed in triplicate, and the data were expressed as the mean ± standard error. Data analysis was performed using an one-way analysis (ANOVA) by Statistical Package for Social Sciences (SPSS) software (version 25.0, IBM, IL, United States). The charts were plotted using Origin 2020. Differences with a *p*-value of < 0.05 were considered statistically significant.

## 3 Results and discussion

### 3.1 The physicochemical properties of BSP

**Figure 1:**
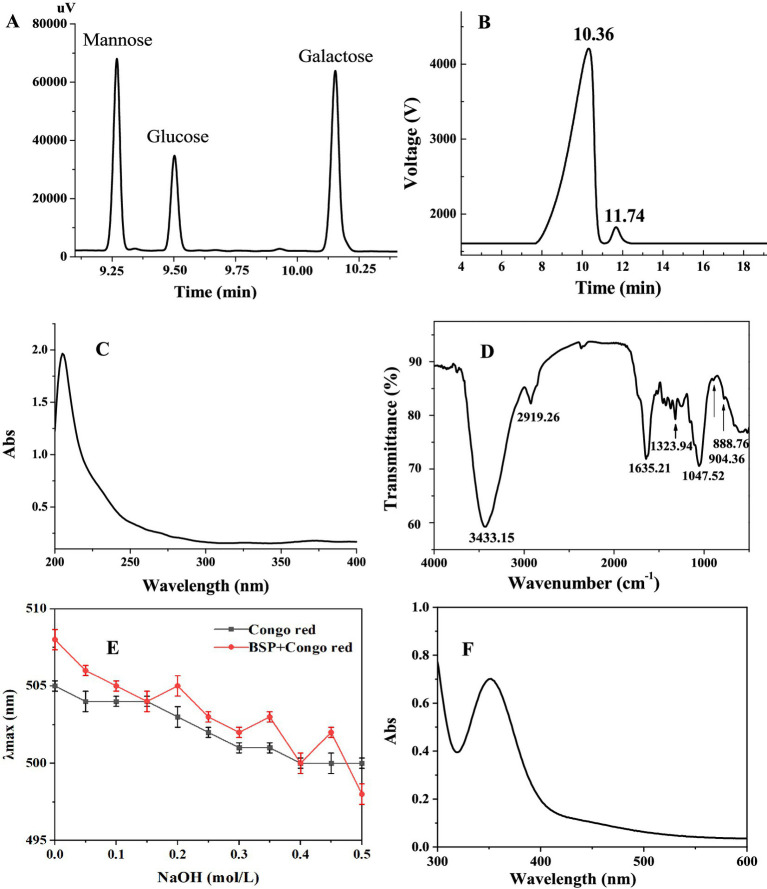
Characterization of BSP. **(A)** Elution profile of BSP hydrolyzate; **(B)** Elution peak on high-performance liquid chromatography (HPLC); **(C)** UV spectrum; **(D)** FT-IR spectrum; **(E)** Congo red experimental result; and **(F)** UV–Vis spectrum in the presence of I_2_–KI.

BSP with high sugar content (88.35 ± 0.12%) was obtained after two-step column purification. No protein and uronic acid were detected. BSP was mainly composed of galactose, mannose, and glucose with a relative molar ratio of 3.2:5.4:1.0 (Figure 1A). The *M*_w_ of BSP was determined to be 85.4 kDa (Figure 1B) based on the retention time of 10.36 min. A small elution peak was found at 11.74 min, indicating that a polysaccharide with an *M*_w_ of 23.2 kDa was contained in BSP. This result was very similar to the findings of Chen et al. (11). They also found that the polysaccharide obtained from *B. striata* was composed of galactose, mannose, and glucose in a molar ratio of 1:2.03:3.45 with a molecular weight of 9.1 × 10^4^ Da.

In addition, it can be observed from the UV spectrum of BSP (Figure 1C) that there is no obvious absorption peak between 260 and 280 nm, confirming the absence of proteins, which is consistent with the result of the chemical method.

### 3.2 Structure characterization of BSP

#### 3.2.1 FT-IR spectrum

Figure 1D showed the FT-IR spectrum of BSP, exhibiting the typical characteristics of polysaccharides in the range of 4,000 – 500 cm^−1^. The strong absorption at 3,433 cm^−1^ was derived from the O−H stretching vibrations (21). The characteristic peaks at 2,919 and 1,323 cm^−1^ were attributed to the stretching and bending vibrations of the C−H bond, respectively (22). The band at 1,635 cm^−1^ was attributed to the −OH flexural vibrations (23). The peaks between 1,200 and 1,000 cm^−1^ reflected the asymmetric glycosidic band vibrations of C−O−C (24). The characteristic absorption bands at 904 and 888 cm^−1^ suggested that both α- and β-configurations existed in BSP (25).

#### 3.2.2 Methylation analysis

The methylated BSP was acid-hydrolyzed and acetylated for the GC–MS analysis. The individual peaks were identified by comparison with the mass spectrum. As shown in Table 1, the results showed the presence of four liberations of 2,3,4,6-Me_4_-Man*p* (residue: 1-linked Man*p*), 2,3,6-Me_3_-Man*p* (residue: 1,4-linked Man*p*), 2,3,6-Me_3_-Glc*p* (residue: 1,4-linked Glc*p*), and 2,3,4-Me_3_-Gal*p* (residue: 1,6-linked Gal*p*). The molar ratio of each monosaccharide (galactose: mannose: glucose) was consistent with the results of HPLC (3.2:5.4:1.0).

**Table 1:** Glycosidic linkage composition of methylated BSP.

Retention time (min)	Methylated sugars	Linkage type	Molar ratio (%)	Major mass fragments (m/z)
10.54	2,3,4,6-Me_4_-Man*p*	1-linked Man*p*	8.56	43, 71, 87, 101, 117, 129, 145, 161, 205
11.25	2,3,6-Me_3_-Man*p*	1,4-linked Man*p*	40.20	43, 87, 101, 113, 117, 129, 143, 161, 173, 233
11.98	2,3,6-Me_3_-Glc*p*	1,4-linked Glc*p*	38.22	43, 87, 101, 113, 117, 129, 143, 161, 173, 233
13.16	2,3,4-Me_3_-Gal*p*	1,6-linked Gal*p*	12.58	43, 87, 99, 101, 117, 129, 161, 173, 189, 233

#### 3.2.3 NMR

**Figure 2:**
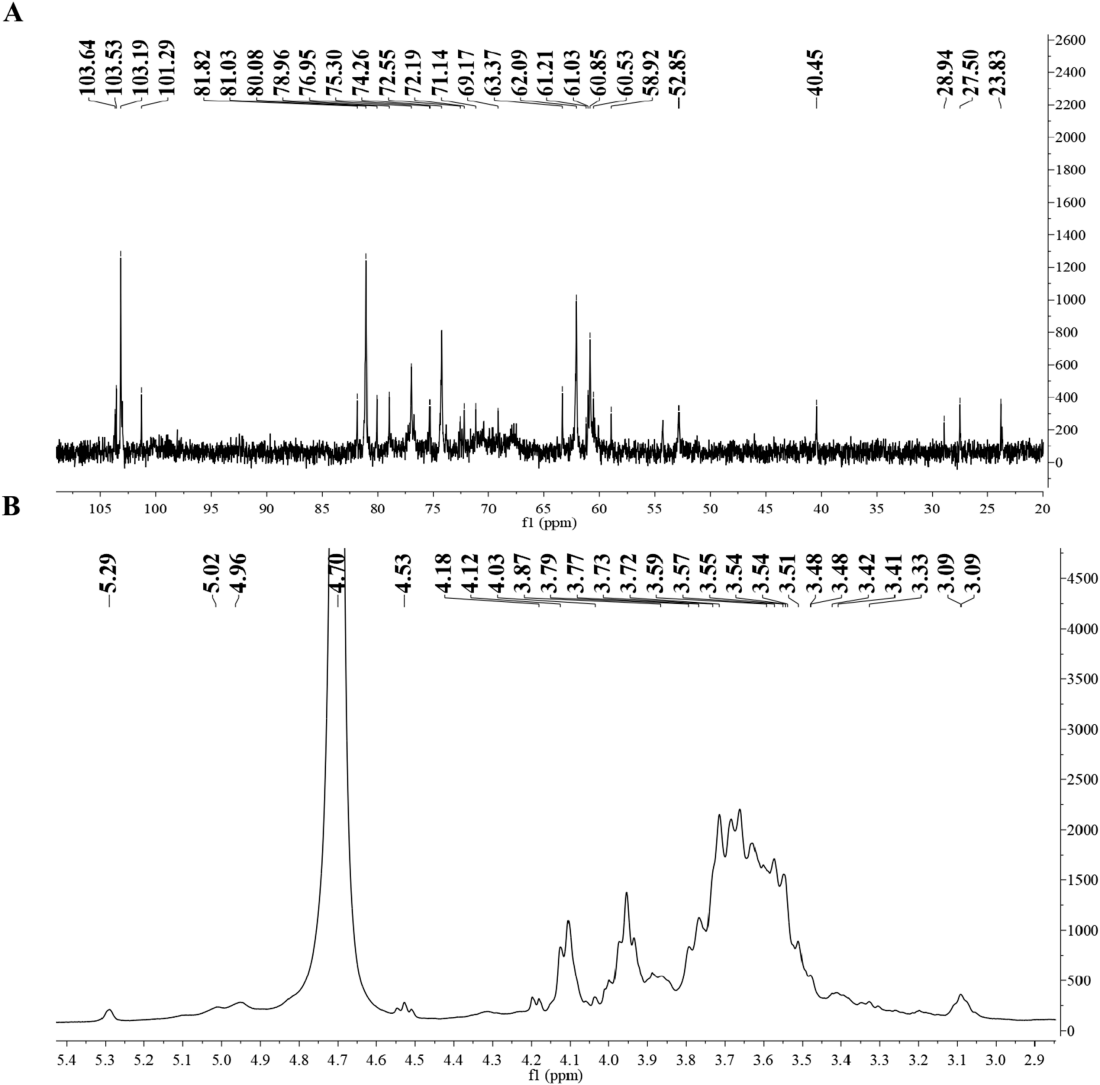
**(A)** ^1^H NMR spectrum of BSP and **(B)** ^13^C NMR spectrum of BSP.

Figures 2A,B showed the ^1^H NMR and ^13^C NMR spectra of BSP; in the ^1^H NMR spectrum, most chemical shifts were concentrated within 3–5 ppm, presenting the typical characteristic of polysaccharides (26). In the ^13^C NMR spectrum, the chemical shifts from 60 to 78 ppm were attributed to the signals of C2–C6 carbons. Moreover, the chemical shifts of anomeric protons (*δ*_H_ 4.0–5.3 ppm) and anomeric carbons (δ_C_ 90.0–110.0 ppm) proved that both α- and β-configurations were present in BSP (27). The peaks accurately occurred at 101.29, 103.19, 103.53, and 103.64 ppm (anomeric carbons) and 5.29, 5.02, 4.96, and 4.53 ppm (anomeric protons), revealing that BSP contained four types of monosaccharide residues. The presence of →4)-β-D-Man*p* -(1 → was proven by the chemical shifts at δ 103.64, 76.95, 71.14, 81.82, 72.55, and 61.03 ppm (C-1–C-6) (19, 28). The main sugar residues of BSP also included →4)-α-D-Glc*p*-(1→, which was confirmed by the signals of *δ*_H/C_ 5.29/101.29, 3.59/72.55, 3.87/72.19, 3.54/78.96, 3.51/69.17, and 3.55/60.85 ppm (29). Similarly, the →6)-β-D-Gal*p*-(1 → and β-D-Man*p*-(1 → were identified based on the corresponding signals. Chen et al. (11) obtained a polysaccharide named pFSP from the fibrous roots of *B. striata* with the *M*_w_ of 9.1 × 10^4^ Da, and pFSP consisted of three monosaccharides, including D-Glc, D-Gal, and D-Man. The main sugar residues in pFSP were →4)-α-D-Glc*p*-(1→, →4)-β-D-Man*p*-(1 → and →3,6)-β-D-Man*p-*(1→, moreover, →6)-β-D-Gal*p*-(1 → and β-D-Man*p*-(1 → were also found in pFSP. Therefore, BSP might have a structure similar to the pFSP, but their *M*_w_s were different.

#### 3.2.4 Congo red and I_2_–KI tests

In the present study, Congo red and I_2_-KI tests were used to determine the spatial configuration of BSP. As shown in Figure 1E, the λmax of BSP mixed with the Congo red solution was the same as that of the Congo red solution alone, and the λmax of two solutions both decreased with the NaOH concentration in a dose-dependent manner. Under different NaOH concentrations, there was no red shift of the λmax in BSP mixed with the Congo red solution, suggesting that BSP had no triple-helix conformation (30).

In addition, the maximum absorption peak of BSP was at 360 nm, there was no absorption peak around 565 nm (Figure 1F), suggesting that long and branched chains were contained in the backbone of BSP (31). This was in accordance with the result of the Congo red test.

#### 3.2.5 SEM and AFM

**Figure 3:**
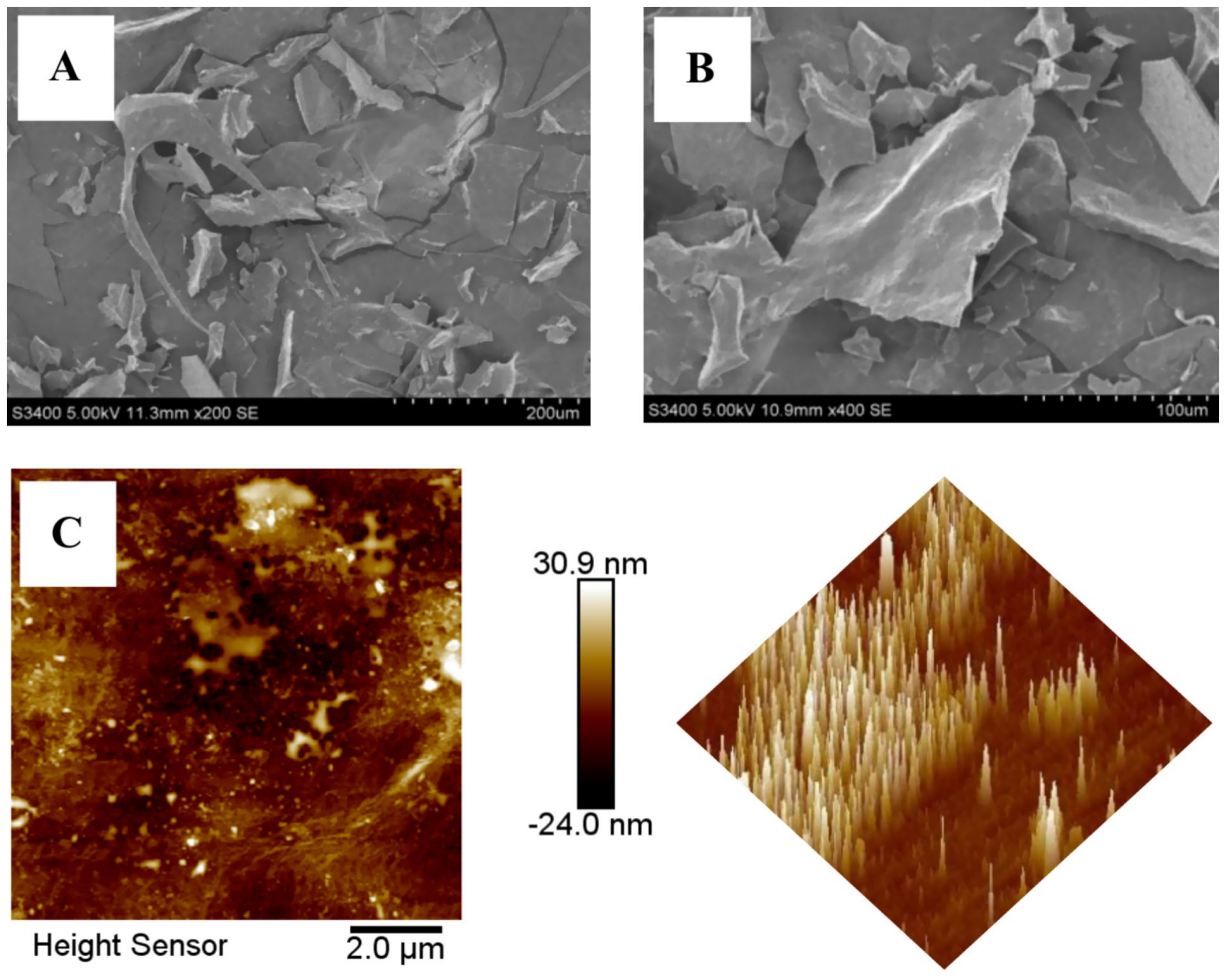
The microscopic surface morphology of BSP **(A)** 200×; **(B)** 400×; **(C)** AFM of BSP.

SEM was used to characterize the microscopic surface morphology of BSP (presented in Figures 3A,B), and the surface exhibited fragmented sheet-like aggregation with a rough surface. Many polysaccharides also showed this feature after lyophilization (19, 32).

AFM exhibited the planar and three-dimensional images of BSP. As shown in Figure 3C, it is clear that BSP was plate-shaped with tight structure, but the lump height (0.5–30.9 nm) was obviously higher than that of individual polysaccharide molecule (<1.0 nm), indicating that the molecules intertwined with each other and formed aggregates due to the hydroxyl groups in the polysaccharide chains.

### 3.3 Inhibition effects of BSP on α-amylase and α-glucosidase

It is well known that α-amylase can hydrolyze α-1,4-glucoside of starch, glycogen, and various maltodextrins and produces oligosaccharides. α-Glucosidase continues to hydrolyze oligosaccharides to produce glucose, which is then absorbed by the intestine, resulting in an increase of blood glucose (2, 33). When these two enzymes were inhibited, the postprandial blood glucose could be reduced (34). Therefore, the bioactive substances with inhibitory abilities against α-amylase and α-glucosidase may have hypoglycemic function.

**Figure 4:**
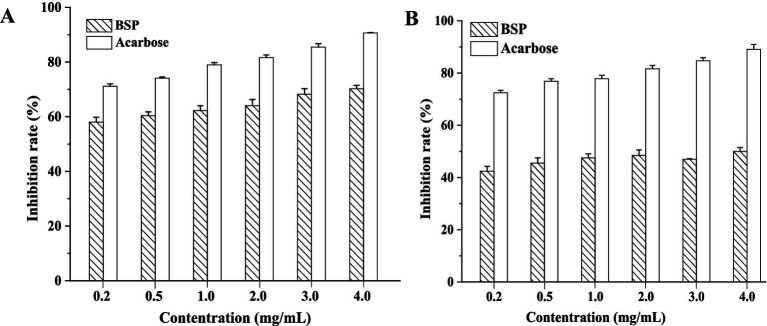
Inhibition of BSP on α-amylase **(A)** and α-glucosidase **(B)**.

Acarbose has been used in clinical trials for the treatment of postprandial hyperglycemia and was chosen for the control in the present study. The inhibition effects of BSP and acarbose on two enzymes are shown in Figure 4. Both BSP and acarbose exhibited concentration-dependent inhibitory activities on the two enzymes within the range of 0–4.0 mg/mL (*p* < 0.05), and at 4.0 mg/mL, the inhibition rates of BSP were 67.75 ± 0.45% against α-amylase and 48.24 ± 1.02% against α-glucosidase. The inhibitory effects of BSP were weaker than those of acarbose under the same conditions. However, the respective half-maximal inhibitory concentration (IC_50_) values of BSP for α-amylase and α-glucosidase were determined as 0.85 and 4.62 mg/mL, which were lower than those of acarbose (0.02 and 0.08 mg/mL). Therefore, the *M*_w_ of BSP was significantly larger than that of acarbose; BSP might show better function than acarbose at the same molar concentration. Fu et al. obtained an acidic heteropolysaccharide (HEP-2) from honeysuckle berries with an *M*_w_ of 3.01 × 10^6^ Da and found that HEP-2 showed strong inhibitory activities against α-amylase and α-glucosidase with IC_50_ values of 3.05 and 1.56 mg/mL, respectively (3). These data were similar to those of BSP. Therefore, BSP had the potential to be used as a hypoglycemic agent for the therapy of diabetes.

It is generally accepted that the polysaccharide molecules could bond with enzyme molecules through hydrogen bonding, leading to a reduction in enzyme activity (35). In addition, the polysaccharides may be adsorbed on the starch or oligosaccharide molecules and hinder their contact with the enzymes, resulting in the inhibition of hydrolysis of starch or oligosaccharide (36).

### 3.4 Inhibition kinetics for two enzymes

**Figure 5:**
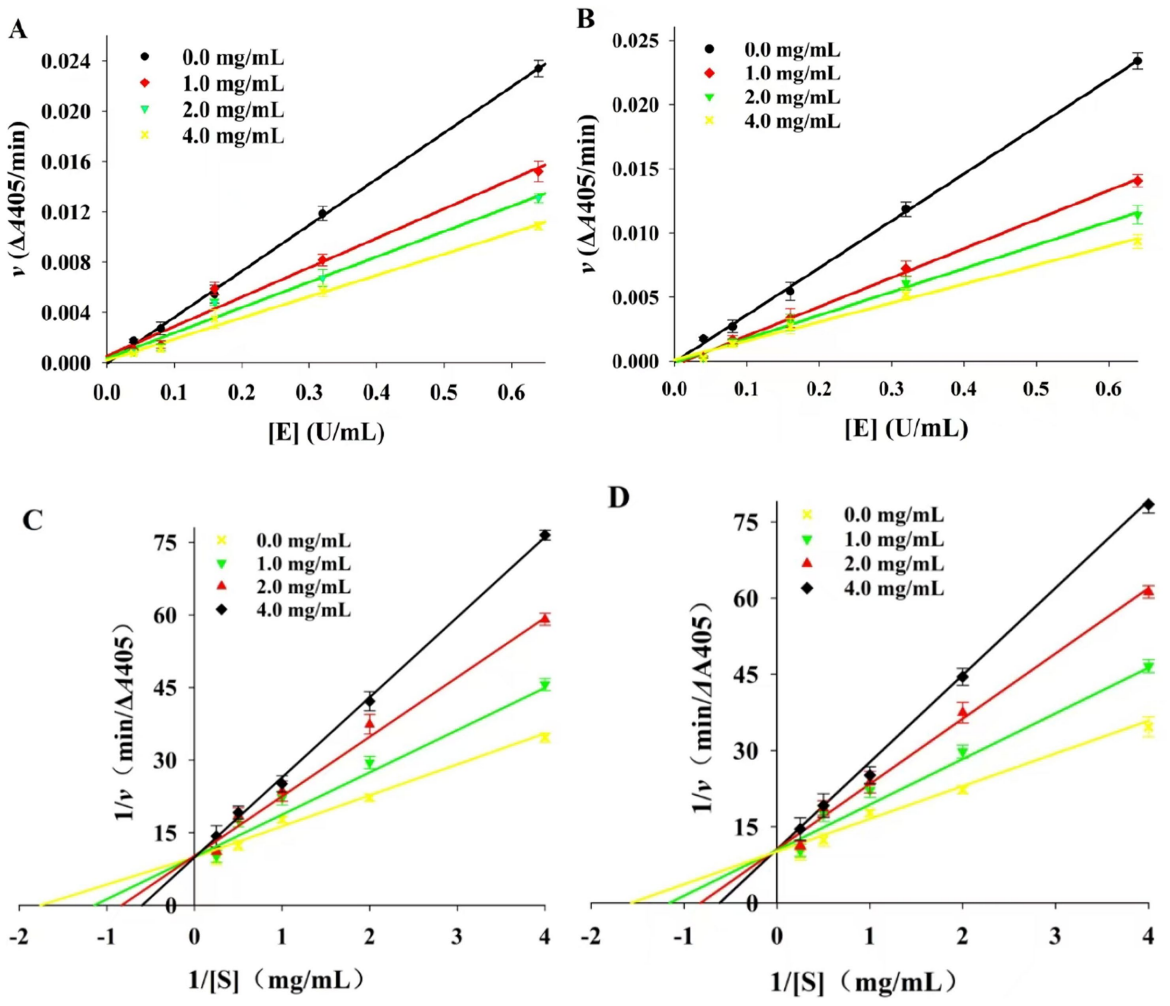
**(A)** Inhibition kinetics curves for α-amylase; **(B)** Inhibition kinetics curves for α-glucosidase; **(C)** Lineweaver-Burk plots of BSP inhibition on α-amylase; **(D)** Lineweaver–Burk plots of BSP inhibition on α-glucosidase.

There are two main types of enzyme inhibition by inhibitors: reversible inhibition and irreversible inhibition. The reaction rate curve of reversible inhibition passes through the origin, and the rate curve of irreversible inhibition does not go through the origin (37). As shown in Figures 5A,B, with different concentrations of BSP, the inhibition kinetic curves of α-amylase and α-glucosidase by BSP were all straight lines and passed through the origin. With increasing BSP concentration, the slopes of these lines decreased, indicating reversible inhibition (38).

The Lineweaver–Burk plots for these two enzymes are shown in Figures 5C,D. The double inverse curves for BSP at different concentrations all converged at one point on the *y*-axis, where the maximum reaction rate *v*_max_ remained constant. At the same time, the slopes of the curves (*K*_m_) increased with the BSP concentration. This increase indicated that the inhibition of α-amylase and α-glucosidase was all reversible competitive types (39). In other words, BSP could bind with enzyme molecules and decrease the activities of two enzymes.

Moreover, the inhibitory constant (*K*_i_) can reflect the ability of the inhibitor to inhibit the enzyme. Generally, the smaller the *K*_i_ value, the better the inhibitory effect (40). The *K*_i_ values for α-amylase and α-glucosidase were 0.28 ± 0.04 and 0.16 ± 0.08 mg/mL, respectively, indicating that binding of BSP to α-glucosidase was more favorable than to α-amylase.

### 3.5 *In vitro* prebiotic activities of BSP

#### 3.5.1 The proliferation of probiotics

**Figure 6:**
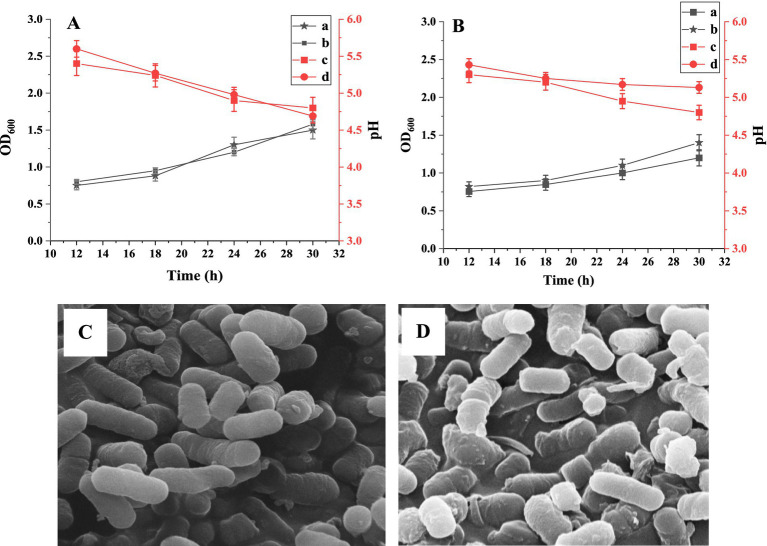
The growth curves of Lactobacillus plantarum **(A)** and Lactobacillus acidophilus **(B)**. **(A)** The OD600 values in BSP medium; **(B)** The OD600 values in glucose medium; **(C)** The pH values in BSP medium; **(D)** The pH values in glucose medium. SEM images of *L. plantarum* **(C)** and *L. acidophilus* **(D)** in BSP medium (×10.0 k).

Using BSP as the sole carbon source, the growth of *L. plantarum* and *L. acidophilus* was monitored, and the pH change of the medium was also tested. As shown in Figure 6A, similar growth curves for *L. plantarum* in BSP and glucose media were obtained. The bacteria numbers increased with the incubation time in two media (*p* < 0.05); the glucose medium had a slightly better effect on bacteria proliferation than the BSP medium. Moreover, the pH values of both culture media decreased with the incubation time (*p* < 0.05), indicating an increase in metabolic activity of the bacteria (41). The increased populations of *L. acidophilus* are shown in Figure 6B, and both glucose and BSP stimulated their proliferation. Based on the pH changes of the media and the increase in bacteria numbers, it could be seen that BSP had a similar effect on the proliferation compared to the glucose at the same concentration.

The proliferation abilities of *L. plantarum* and *L. acidophilus* in BSP media were characterized using SEM, as shown in Figures 6C,D. Both bacterial strains exhibited full and complete morphology, indicating a good reproductive state.

#### 3.5.2 The production of SCFAs

The intestinal microbiota produces a variety of small molecular acids through the fermentation of dietary fiber and other substrates, mainly including SCFAs and some other metabolites. SCFAs, such as acetic acid, propionic acid, and butyric acid, are not only essential for intestinal health but also have a profound impact on systemic health (42).

Table 2 shows the production of SCFAs in two media. It was found that acetic acid, propionic acid, and butyric acid were generated after a 30-h incubation; this well-explained the decrease in pH values. The concentrations of total SCFAs in glucose and BSP media were very similar, indicating the proliferation of probiotics and the conversion of BSP into SCFAs. After considering *L. plantarum* as an example, the concentrations of acetic acid, propionic acid, and butyric acid in the BSP medium were 122.64 ± 0.14, 88.15 ± 0.46, and 175.32 ± 0.34 μmol/L, respectively. In contrast, these concentrations were 134.72 ± 0.68, 85.66 ± 0.36, and 172.85 ± 0.18 μmol/L in the glucose medium. Lee et al. (43) studied the prebiotic activity of mucilage polysaccharide from molokhia leaves (MPF) with an *M_w_* of 51.2 kDa. They discovered that MPF increased the contents of acetic acid, propionic acid, and butyric acid, which correlates with our findings. These results further demonstrated that BSP exhibited prebiotic activity and could serve as a carbon source to stimulate the reproduction of probiotics.

**Table 2:** Short-chain fatty acid profile in glucose and BSP media.

Bacteria	Carbon sources	Acetic acid (μmol/L)	Propionic acid (μmol/L)	Butyric acid (μmol/L)
Lactobacillus plantarum	BSP	122.64 ± 0.14	88.15 ± 0.46	175.32 ± 0.34
	Glucose	134.72 ± 0.68	85.66 ± 0.36	172.85 ± 0.18
Lactobacillus acidophilus	BSP	132.08 ± 0.20	74.58 ± 0.28	162.36 ± 0.26
	Glucose	130.45 ± 0.14	72.78 ± 0.42	170.55 ± 0.15

## 4 Discussion

Recently, *B. striata* polysaccharides (BSPs) have attracted much attention from industries and researchers due to their remarkable immunomodulatory, antioxidant, anticancer, hemostatic, anti-inflammatory, antimicrobial, gastroprotective, and liver-protective effects (6–13). It was recognized that the bioactivities of polysaccharides were connected to their monosaccharide composition, *M*_w_, chemical composition, glucosidic bond, conformation, and so on (44). To date, a variety of BSPs have been purified and identified; the different raw materials, extraction, and purification methods lead to differences in the chemical composition and structural characteristics of BSPs, thus leading to various biological activities (45). Several studies have demonstrated that the *M*_w_ of BSPs is tightly linked to their biological activities; however, the influencing rule is inconsistent. The low-*M*_w_ BSPs have shown more remarkable antitumor activities than high-*M*_w_ BSPs (46). However, high-*M*_w_ BSP-1 (83.54 kDa) exhibited more potent immunomodulatory effects than BSP-2 (12.60 kDa) (47). Moreover, pBSP exhibited a potential protective effect against H_2_O_2_-induced injury in fibroblast cells, which was possibly associated with its higher *M*_w_ (48).

To our knowledge, this is the first report on the use of BSP for digestive enzyme inhibition and prebiotic effects. Due to the limited literature on the chemical structures of BSPs, elucidating the structure–activity relationship (SAR) of BSPs is temporarily impossible. Undoubtedly, a comprehensive understanding of SAR would advance the applications of BSP-based dietary supplements and therapeutic medications. Therefore, further scientific research is urgently required to shed light on the SAR of BSPs.

## 5 Conclusion

In this study, a polysaccharide named BSP was isolated from *B. striata* with the sugar content of 88.35 ± 0.12%. BSP consisted of galactose, mannose, and glucose in a molar ratio of 3.2:5.4:1.0. BSP exhibited significant inhibitory effects on α-amylase and α-glucosidase and was a competitive inhibitor for these two enzymes. The *K*_i_ values for α-amylase and α-glucosidase were 0.28 ± 0.04 and 0.16 ± 0.08 mg/mL, respectively. BSP could be used as a carbon source to stimulate the reproduction of probiotics. This study is also the first to investigate the hypoglycemic effect and prebiotic activity of BSP. This study provides a basis for further study on hypoglycemic activity and gastrointestinal health *in vivo.*

## Data Availability

The original contributions presented in the study are included in the article/supplementary material, further inquiries can be directed to the corresponding author.
